# Cohort cholecystectomies in the Brazilian public system: is access to laparoscopy universal after three decades?

**DOI:** 10.1590/0100-6991e-20223180-en

**Published:** 2022-07-08

**Authors:** JOSÉ GUSTAVO OLIJNYK, ISABELLE GARIBALDI VALANDRO, MARCELA RODRIGUES, MAURO ANTÔNIO CZEPIELEWSKI, LEANDRO TOTTI CAVAZZOLA

**Affiliations:** 1 - Hospital Nossa Senhora da Conceição (HNSC), Serviço de Endocrinologia Clínica e Cirúrgica - Porto Alegre - RS - Brasil; 2 - Hospital Militar de Área de Porto Alegre (HMAPA), Serviço de Cirurgia - Porto Alegre - RS - Brasil; 3 - Universidade Federal do Rio Grande do Sul (UFRGS), Faculdade de Medicina - Porto Alegre - RS - Brasil; 4 - Universidade Federal do Rio Grande do Sul (UFRGS), Departamento de Endocrinologia - Porto Alegre - RS - Brasil; 5 - Universidade Federal do Rio Grande do Sul (UFRGS), Departamento de Cirurgia - Porto Alegre - RS - Brasil

**Keywords:** Public Health Practice, Cholecystectomy, Laparoscopic, Health Management, Sistema Único de Saúde, SUS, Saúde Pública, Colecistectomia Laparoscópica, Sistema Único de Saúde, Gestão de Ciência, Tecnologia e Inovação em Saúde

## Abstract

**Objective:**

videosurgery in Brazil started in 1990 with the performance of laparoscopic cholecystectomy, being included by the public health system in 2008. We evaluated the current situation of the use of this technology in the Unified Health System (SUS - Sistema Único de Saúde).

**Methods:**

from 2013 to 2019, 1,406,654 patients registered at the SUS Informatics Department (DATASUS) were analyzed to calculate the rate of laparoscopic cholecystectomies (LC) in relation to open cholecystectomies (OC). Patient characteristics, disease presentation and postoperative mortality were evaluated.

**Results:**

the LC rate reached 41.5% (growth of 68%) with no decrease in the absolute number of OC. In University Hospitals (UH), the LC rate reached 91.96%. The open technique in emergencies was more associated with male patients, aged 60 years or older, with prolonged hospitalization and in the ICU. Those undergoing LC were less predisposed to postoperative death, both electively (OR 0.49; 95% CI 0.42 - 0.56; NNT=20) and urgently (OR 0.23; 95% CI 0.20 - 0.25; NNT ≅1), providing a protective effect.

**Conclusion:**

despite the increase in the indication of LC, the open technique during the years studied remained stable and the most used in the public health system in Brazil. The effectiveness of public health policies to shorten the complete implementation of videosurgery in SUS needs to be investigated in future epidemiological studies, as well as its impact on postoperative morbidity and mortality.

## RESUMO

**Introdução:**

a videocirurgia no Brasil iniciou em 1990 com a realização da colecistectomia laparoscópica, sendo incluída pelo sistema público de saúde em 2008. Avaliamos a situação atual do emprego desta tecnologia no Sistema Único de Saúde (SUS).

**Métodos:**

de 2013 a 2019, 1.406.654 pacientes registrados no Departamento de Informática do SUS (DATASUS) foram analisados para calcular a taxa de colecistectomias laparoscópicas (CL) em relação a colecistectomias abertas (CA). Avaliaram-se características dos pacientes, apresentação da doença e mortalidade pós-operatória.

**Resultados:**

a taxa de CL atingiu 41,5%, com crescimento de 68%, sem ocorrer diminuição do número absoluto de CA. Já em Hospitais Universitários (HUs) a taxa de CL chegou a 91,96%. A técnica aberta em urgências esteve mais associada a pacientes masculinos, com 60 anos ou mais, à internação prolongada e em UTI. Aqueles submetidos à CL estiveram menos predispostos à morte pós-operatória, tanto em caráter eletivo (OR 0,49; IC 95% 0,42 - 0,56; NNT = 20) como na urgência (OR 0,23; IC 95% 0,20 - 0,25; NNT ≅ 1), conferindo efeito protetor.

**Conclusão:**

apesar do aumento da indicação da CL, a cirurgia aberta durante os anos estudados se manteve estável e a técnica mais utilizada no sistema público de saúde do Brasil. A efetividade de políticas de saúde pública para abreviar a completa implementação da videocirurgia no SUS necessita ser investigada em estudos epidemiológicos futuros, assim como seu impacto na morbimortalidade pós-operatória.

## INTRODUCTION

The acceptance of laparoscopy was boosted after successful procedures such as appendectomy, in 1980 (Kurt Semm - Germany) and cholecystectomy, in 1985 (Eric Mühe, Germany)^1^ ^,^ ^2^. In Brazil, laparoscopic cholecystectomy (LC) started in 1990 (Thomas Szego, São Paulo)^3^ and, in 2008, it was made available in the Public Unified Health System (SUS - Sistema Único de Saúde)^4^ ^,^ ^5^. Since then, in this sector, LC for the treatment of cholelithiasis (prevalence in 9.3 - 10.3% of the Brazilian population)^6^ ^,^ ^7^ is the most frequent laparoscopic procedure^8^ ^,^ ^9^. In 2012, its rate was 24% in relation to open cholecystectomy (OC)^8^. In 2008, LC was included by the National Supplementary Health Agency (ANS) as a mandatory procedure to be made available in private health plans^10^, which cover 24.5% of the Brazilian population^11^. In 2010, its rate was estimated at 90% of cholecystectomies^12^.

We analyzed the evolution of laparoscopic cholecystectomies in SUS, according to the characteristics of the patients and the disease presentation. Considering the 127.5% increase in LCs between 2008 and 2012^8^, we aimed to confirm the hypothesis of maintenance of this trend in the subsequent seven-year period, associated with the analysis of hospital information, and its impact on postoperative morbidity and mortality. 

## METHODS

On March 17, 2021, we accessed to SUS Informatics Department (DATASUS), and transferred data files^13^, including the TabWin415 Program of SUS Hospital Information System (SIHSUS). We used data from Hospital Admission Authorizations (AIH Reduzidas) - a system used by the Ministry of Health that identifies the procedure performed during hospitalization. LC was set as the main indicator of the use of laparoscopic surgery in Brazil^8^ ^,^ ^9^ in a retrospective cross-sectional study, from January 2013 to December 2019, with the geographic coverage of the 27 Federation units.

We selected the procedures cholecystectomy (407030026) and laparoscopic cholecystectomy (0407030034) and the type of admission (elective or emergency) in all Brazilian hospitals and in University Hospitals (UHs), linked to the Ministry of Education (MEC). We excluded cholecystectomies due to oncologic reasons. Hospital information included: sex; age group; diagnose (ICD) - cholelithiasis and cholecystitis (K80.0; K80.1; K80.2), acute biliary pancreatitis (K85.1), calculus of bile duct (K80.3; K80.4; K80.5), approved AIH and total value, length of hospital and ICU stays, mortality rate, number and causes of deaths.

### Statistical analysis

We analyzed the association of categorical variables and surgical technique with the chi-square test and Odds Ratio (OR), with a confidence interval of 95% and p<0.05 for statistical significance. To verify the consistency of the DATASUS information, we calculated the trend curve of the expected increase in LC for 2019, based on the growth found between 2008 and 2012^8^. This estimate was obtained through linear regression, whose intraclass correlation coefficient between the increasing trend and the observed was 0.97. Afterwards, in view of the percentage increase in the period, we projected when the rate of laparoscopic surgery would reach 90% of the cases. We used the IBM SPSS Statistics 20.0 software.

### Ethical aspects

DATASUS is in the public domain, in accordance with the Brazilian Access to Information, Privacy, and Health Research Act, nº 12,527/2011^14^. So, there was no need to forward the project to the Ethics in Research Committee.

## RESULTS

In the period studied, there were 1,795,422 admissions in SUS for cholelithiasis and cholecystitis. Of these, 78% underwent cholecystectomy, generating a cost of R$ 786,041,428.30 (US$ 248,747,287.40)^15^. UHs contributed with 3.7% of the total and, in 2017, using the laparoscopic route in more than 90% of cases; in 2019, they reached 91.96%. The laparoscopic rate found in 2019 in SUS hospitals in general was 41.5%, with a growth of 68% when compared to 2012 (24,7%)^8^.

**Figure 1:**
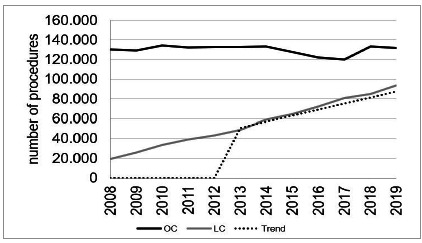
Laparoscopic and open cholecystectomies in SUS (2008 - 2019) ^13^ .

There was an increase in the absolute number of procedures in the period, with 182,008 cholecystectomies performed in 2013 and 225,698 in 2019 (increase of 24%). Elective LC represented the segment with the highest absolute growth, with 37,229 patients operated in 2013 and 70,674 in 2019 (89.83% increase). Despite the lower number, emergency LC showed an even more expressive percentage increase, of 94.96%, with 11,827 patients in 2013 and 23,058 in 2019. The stabilization of the total number of open cholecystectomies performed in 2013 and 2019 (132,952 and 131,966: 1% reduction) is noteworthy. On an elective basis, OC showed an increase of 0.03%, and in emergency, a reduction of 3.56% (Figure 1).

In view of the real increase in the laparoscopic rate and comparing it with the expected trend curve, we observe the similarity, as shown in Figure 1. In 2013, the rate was 27%, while 28% was expected, and this behavior continued in subsequent years. Following this trend of increase in laparoscopic procedures and accepting the hypothesis of OC reduction in the same proportion (considering stabilization in the same number of procedures in 2019), we projected that a 90% LC rate would be reached in SUS general hospitals only in 2037.

### Differences Between Sexes

Females accounted for 80% of the cases, with a 16% greater chance that the procedure would be open in an emergency when compared with elective surgery (OR 0.86, 95% CI 0.85-0.87, p<0.0001). Male patients had a 6% greater chance that the laparoscopic access would be used in elective surgeries; in urgent cases, there was an increase of 6% in the open technique. In UHs, there was a greater chance of indication of laparotomy in male individuals, both in elective (25% increase) and in urgent cases (66% increase) (Table 1). Even so, when considering all hospitals in Brazil, male patients were more likely to undergo the laparoscopic technique: in electives, OR 1.06 (95% CI 1.03-1.08, p<0.0001), and in emergencies, OR 1.07 (95% CI 1.02-1.12, p=0.001) .

**Table 1:** Cholecystectomies according to the type of access and hospitalization in Brazilian regular and University hospitals (2013 - 2019) ^13^ .

	Brazil - non-UH			University Hospitals - MEC		
Variable (%)	Vídeo	Open	OR	Vídeo	Open	OR
	n=504,917	n=901,737	(95% CI)*	n=49,574	n=8,758	(95% CI)*
Age ≥60 years						
Elective	23.52	20.59	1.18 (1.17 - 1.19)	23.91	26.17	0.88 (0.83 - 0.94)
Emergency	22.14	23.68	0.91 (0.90 - 0.93)	22.97	34.64	0.56 (0.51 - 0.62)
Male Sex						
Elective	18.48	17.55	1.06 (1.05 - 1.07)	19.28	22.96	0.80 (0.75 - 0.85)
Emergency	23.83	24.93	0.94 (0.92 - 0.95)	25.04	35.73	0.60 (0.54 - 0.66)
Urgent Procedure	25.00	28.29	0.84 (0.83 - 0.85)	22.90	25.80	0.85 (0.80 - 0.89)
Discharge <24 hours	2.18	0.52	4.23 (4.06 - 4.40)	0.59	0.38	1.53 (1.01 - 2.31)†
Discharge ≥72 hours						
Elective	12.57	22.89	0.48 (0.47 - 0.49)	19.71	40.11	0.36 (0.34 - 0.38)
Emergency	51.92	52.98	0.95 (0.94 - 0.97)	58.19	74.00	0.48 (0.44 - 0.53)
ICU Admission						
Elective	1.16	0.75	1.54 (1.48 - 1.61)	0.82	3.48	0.22 (0.19 - 0.27)
Emergency	2.84	4.27	0.65 (0.63 - 0.68)	2.33	9.72	0.22 (0.18 - 0.26)
Mortality Rate						
Elective	0.07	0.14	0.49 (0.42 - 0.56)	0.08	0.74	0.10 (0.06 - 0.16)
Emergency	0.33	1.43	0.23 (0.20 - 0.25)	0.25	4.28	0.05 (0.03 - 0.08)

OR, Odds Ratio (confidence interval 95%); *p value <0.001; † p value = 0.41. Source: Ministry of Health - Brazil. 1,406,654 patients.

### Age Group

**Figure 2:**
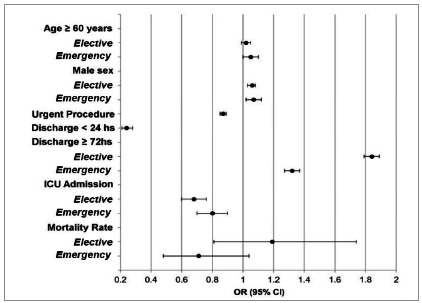
Factors related to the performance of laparoscopic cholecystectomy in UHs (MEC) versus other hospitals in Brazil ^13^ .

In patients aged ≥ 60 years, elective LC was performed with a chance greater than 18% compared with the open approach, with a 9% higher chance of laparotomy in emergencies. In the care of this same group in UHs, the reduction of the laparoscopic route was 12% in elective surgeries and 44% in emergencies. When comparing the two places of care, there was no difference in the indication of the type of access for patients aged ≥60 years, regardless of disease presentation. Without a specific age group discrimination, in both UHs and non-UHs we observe that emergency cases favored open surgery, with an increase of 19% and 17%, respectively. In addition, UHs were 13% less likely to have laparoscopic access than other hospitals in this scenario (Figure 2).

### Hospital Stay

Laparoscopy favored hospitalizations with less than 24 hours (supposedly elective), despite the low occurrence in UHs, 76% lower than that practiced in other hospitals. There was an increase in prolonged hospitalizations (≥72h) in emergencies, with a 52% reduction in their need for elective LC; in UHs this reduction occurred in 64%. However, when compared with Brazil in general, the chance of prolonged hospitalization was higher in UHs undergoing laparoscopic surgeries, both elective and urgent (Figure 2).

### Admission to the Intensive Care Unit (ICU)

There was a 45% reduction in the chance of needing ICU care when laparoscopy was used in the emergency setting compared with the open technique. In elective surgeries, however, we observed an opposite trend, with an increase of 54%. In UHs, there was a 78% reduction in the need for ICU in LC in both forms of disease presentation. When compared with other hospitals, it was 32% less likely in elective surgeries and 20% in emergencies (Figure 2).

### Mortality rate

From 2013 to 2019, there were 5,207 deaths within 30 days after the procedure. Compared with OC, LC showed a 51% reduction in the chance of death in elective surgeries and in 77% in emergencies. In UHs, we identified an even greater reduction, of 90% and 95%, respectively. There was no difference in mortality of patients operated by laparoscopy between these two realms of care. However, when OCs are analyzed, the mortality rate in UHs proved to be higher than in other emergency hospitals, with an OR of 3.13 (95% CI 2.55-3.85, p<0.0001). 

The mortality rate of the elective presentation was 0.11% in OC and 0.06% in LC. In emergencies, it was 1.43% for OC and 0.33% for LC. When comparing by sex and in patients aged ≥60 years, we found that men in this age group were 2.04 times more likely to die than women in elective OC (95% CI 1.74-2.39, p<0.0001) and 1.54 times in urgent ones (95% CI 1.43-1.66, p<0.0001). In elective LC, there was no difference in the risk of death between sexes, with OR 1.37 (95% CI 1.00-1.88, p=0.04). In the case of emergency LC, the chance was 1.45 times higher for men (95% CI 1.16-1.83, p=0.0001). When studying the chance of death between sexes separately (≥60 years), we observed that in men, open surgery has an elective OR of 3.05 (95% CI 2.29-4.07, p<0.0001), and in urgency, 4.53 (95% CI 3.78-5.43, p<0.0001). In females, elective OC presented an OR of 2.05 (95% CI 1.67-2.52, p<0.0001), and in urgency, 4.26 (95% CI 3.64-4.99, p<0.0001).

Between 2013 and 2014 (data not available in the other years of the series), there were 1,515 deaths, with mortality rates similar to those of the entire period in electives (OC 0.15 versus 0.11; LC 0.09 versus 0.06) and in emergencies (OC 1.46 versus 1.43; LC 0.37 versus 0.33). When we adjusted the analysis by the two groups with the highest representation in death causes (Digestive System Diseases; Neoplasms and Diseases of the Circulatory System), there was no difference, both for the presentation of the disease and in the form of intervention for cause of death in OC versus LC: in emergencies, OR 0.64 (95% CI 0.32-1.28, p=0.20); and in electives, OR 0.88 (95% CI 0.44-1.73, p=0.70). In the group of patients who died with associated choledocholithiasis, there was no association by age group (above and below 60 years), both in UHs, OR 0.30 (95% CI 0.03-2.42), and in other hospitals, OR 0.30 (95% CI 0.03-2.42). The same occurred in the comparison between sexes over 60 years old, OR 0.50 (95% CI 0.26- 0.95, p=0.34).

## DISCUSSION

The availability of videosurgery in SUS as a technology of feasible and routine use for the treatment of cholelithiasis resulted in an increase in the absolute number of patients operated, as previously reported^16,17,^ significantly mobilizing resources. However, its adoption was wider in patients treated by private health insurance plans, not yet accompanied by the public system. In 2019, 58.5% of cholecystectomies were still performed using the open technique, and in emergencies this percentage rose to 66.9% (world data report up to 48.7%)^18^. In Brazilian UHs, we found 16.63% of such procedures.

Despite the growth in the use of the laparoscopic technique in SUS since 2013, there was no drop in the same proportion in open surgeries, which remained stable. In addition, the increase in the incidence of hospital admissions with diagnoses of symptomatic cholelithiasis and acute cholecystitis did not follow the same percentage increase in procedures: 24% in elective admissions versus 89.83% in LC; and 8.4% in emergency admissions versus 94.96% in LC. Possibly, laparoscopy has increased the number of patients with surgical indication, both for those who would previously only be treated symptomatically and for those who would have the acute process “cooled down” with clinical treatment.

In the UHs, this change occurred more sharply during the first five years of the survey, since in 2017 they reached the laparoscopy rate found in 2010 in the private health plans’ services. One of the factors that could be involved in this transformation is the National Restructuring Program for Federal University Hospitals (Rehuf), coordinated by the Brazilian Hospital Services Company (Ebserh) and executed in partnership with the Ministries of Health and Education. In 2013, R$ 82.4 million were invested in hospital services, with the acquisition of videosurgery equipment for 29 federal UHs in all Brazilian regions^19^.

### Hospitals in Brazil

By controlling for patient characteristics, hospitalization and disease presentation we were able to determine their impact on the performance of one of the access techniques. We identified that men and patients aged ≥60 years were more likely to undergo open surgery in emergencies, in addition to being more prone to prolonged hospitalization and the use of ICU beds. This behavior follows the trend of published data, but with lower percentages of the open technique (3.7% in young people and 9.2% in the elderly)^20^. Contrary to the concept of greater aesthetic benefit that laparoscopic surgery offers to women, men were more likely to undergo elective treatment through this route; however, emergencies led to the use of the open technique. Additionally, as expected, prolonged hospital stay is more associated with open and emergency surgeries; laparoscopy is associated with shorter stay, even in emergencies, as previously reported^21^. 

A relevant finding of this study was the need for ICU beds. As expected, there was a greater chance of ICU admission in cases of emergency surgery, especially open ones; in elective laparoscopic surgeries, however, when the use of this resource was not expected, there was a higher rate than in elective open surgeries. Aiming to understand this event, in a reanalysis by age groups in elective LC, we observed that patients aged ≥60 years were six times more likely to be admitted to the ICU than younger ones, OR 6.00 (95% CI 5.64-6.39; p<0.0001). Possibly, these older patients, despite being considered suitable for elective surgery in the preoperative evaluation, because of the surgical trauma had some degree of decompensation of comorbidities, which are more frequent in this population. In a study of elective cholecystectomies in octogenarians, the reported need for ICU admission was 31%^22^. In the present study, 31.7% of patients aged 60 years or older needed to be admitted to the ICU when undergoing elective LC. In younger patients, the rate was 0.54%.

### University Hospitals

Although 91.96% of the cases in UHs were operated by laparoscopy in 2019, we identified some particularities in the use of this surgical access when adjusted for the characteristics of interest and compared to other hospitals. We found no difference in the indication of LC between the two spheres of care in patients ≥60 years, regardless of disease presentation, despite a greater tendency towards laparoscopy for males in UHs. Surprisingly, in these institutions there was a lower rate of hospital discharge less than 24 hours after elective procedures, despite the already demonstrated safety^23^, and higher rate of prolonged hospitalizations. Notwithstanding the greater propensity to use ICU beds in Brazilian hospitals in general, there was no difference in postoperative mortality in the laparoscopic group between the two types of institution.

### Postoperative Mortality

According to PAHO/WHO data, “[...] assuming a 3% rate of perioperative adverse events and a 0.5% rate of global mortality, almost seven million surgical patients will have significant complications each year, of whom one million will die during or immediately after surgery”^35^. Patients operated by the open technique had the highest death rates, particularly in emergencies, in both sexes. In UHs, there was a 3.13 times greater chance of death in emergency OC when compared with the other hospitals, in addition to a greater propensity to use the open route. As UHs are more complex reference centers, they may have attended to the most severe presentations of the disease or those with worse clinical conditions. A possibility (refuted by the lack of association with high age and gender) would be the higher incidence of bile duct exploration due to choledocholithiasis or even indication of subtotal cholecystectomy in critically ill patients^24^. In such scenarios, the open route could have been indicated, with an impact on increased mortality, as reported in pre-laparoscopic series^25^ ^,^ ^26^.

Mortality in open surgeries was higher in male patients. We did not find this association in elective laparoscopic surgeries, which demonstrates its protective factor, also evidenced in the lower chance of death with LC than with OC in the emergency setting.

In view of the reduction in the attributable risk of death in the group treated by laparoscopy regardless of disease presentation, we can establish that the number necessary to be operated on by this technique to avoid death occurring in OC was three patients (NNT=2.7). If we consider elective surgeries, this number was 20 LC. For emergency cases, the calculated NNT was ≅1 (0.91), that is, every patient would benefit from the use of laparoscopy, as each LC performed would prevent one death occurring in the postoperative period of open surgery. To allow for comparison of the strength of this intervention, we cite the ISIS-2 study^27^, which evaluated the combined use of streptokinase and aspirin in patients with acute myocardial infarction, finding an NNT of 19.

The projection of the trend curve to reach 90% in the rate of laparoscopic surgery estimated that another 18 years would be needed after the end of the study period, with a concomitant reduction in OCs, accepting the hypothesis of maintenance of the number of procedures performed in 2019 in subsequent years. If it were possible to adopt this rate immediately from January 2020, maintaining the differences found in the mortality rates between the two techniques, by 2037 2,795 deaths would have been avoided (estimate of 6,417 deaths in OC and 3,622 deaths in LC from 2020 to 2037).

### Limitations and Bias Control

DATASUS depends on the completion of forms in the performing hospitals, with the possibility of information bias, as in the specification of the access route. However, when analyzing the trend curve (Figure 1), the projection of the number of LCs that would be expected until 2019 followed the actual data, suggesting that the procedures are being correctly identified.

We found insufficient information in DATASUS about characteristics such as clinical comorbidities, severity classification of acute cholecystitis^28^, early or late indication for surgery^24^, method of treating choledocholithiasis, bile duct injury (estimated at up to 1.5% in LC and 0.2% in OC)^29^ ^,^ ^30^, laparotomy conversion rates (estimated at 4.2%-6.2%)^31^, and reintervention. This made it impossible to fully adjust the groups for multivariate logistic regression analysis. Therefore, the reduced chance of postoperative death attributed to laparoscopy may have been overestimated. On the other hand, reviewing surgical series published before and after the implementation of laparoscopic surgery worldwide, mortality rates in cholecystectomies range from 0.05% to 4.1%, depending on the access technique and degree of severity of cholecystitis^16^ ^,^ ^25^ ^,^ ^26^ ^,^ ^31^ ^-^ ^34^, similar to our findings. Additionaly, there was no difference in deaths in 2013 and 2014 when neoplastic and cardiovascular comorbidities were present, especially in emergency open surgeries, which mortality rate was 3.94 times higher than that of laparoscopy. 

Given the restriction of elective surgeries in Brazilian hospitals due to the SARS-CoV-2 pandemic, we excluded 2019 from the analysis, since we found a 51% reduction in elective cholecystectomies in a parallel survey conducted in DATASUS. The repercussion of this pileup of surgeries will possibly be the increase in the share of emergency surgeries, which from 2013 to 2019 represented 27% of all procedures.

## CONCLUSION

The LC rate evidenced the limited access to technology in the Brazilian public health system and its impact on postoperative mortality. Although the risk reduction conferred by the laparoscopic technique may have been overestimated in emergencies due to limited information from DATASUS, the possibility of an increased chance of death due to the use of the open technique in elective cholecystectomies makes its indication unjustifiable nowadays.

We believe that results such as ours can support actions by Brazilian medical entities to stimulate government policies for the implementation and popularization of technologies in SUS, with a significant impact on morbidity and mortality.
